# Is there A Role for Alpha-Linolenic Acid in the Fetal Programming of Health?

**DOI:** 10.3390/jcm5040040

**Published:** 2016-03-23

**Authors:** Alicia I. Leikin-Frenkel

**Affiliations:** 1 The Sackler Faculty of Medicine, Tel Aviv University, Tel Aviv 69978, Israel; alicial@post.tau.ac.il; Tel.: +972-353-29-39; Fax: +972-030-530-4431; 2 Bert Strassburger Lipid Center, Sheba, Tel Hashomer, Ramat Gan 52621, Israel

**Keywords:** alpha linolenic acid, maternal diet, fetal programming, metabolic syndrome, gene expression, ω3 fatty acids

## Abstract

The role of ω3 alpha linolenic acid (ALA) in the maternal diet during pregnancy and lactation, and its effect on the prevention of disease and programming of health in offspring, is largely unknown. Compared to ALA, ω3 docosahexaenoic (DHA) and eicosapentaenoic (EPA) acids have been more widely researched due to their direct implication in fetal neural development. In this literature search we found that ALA, the essential ω3 fatty acid and metabolic precursor of DHA and EPA has been, paradoxically, almost unexplored. In light of new and evolving findings, this review proposes that ALA may have an intrinsic role, beyond the role as metabolic parent of DHA and EPA, during fetal development as a regulator of gene programming for the prevention of metabolic disease and promotion of health in offspring.

## 1. Introduction

**Figure 1:**
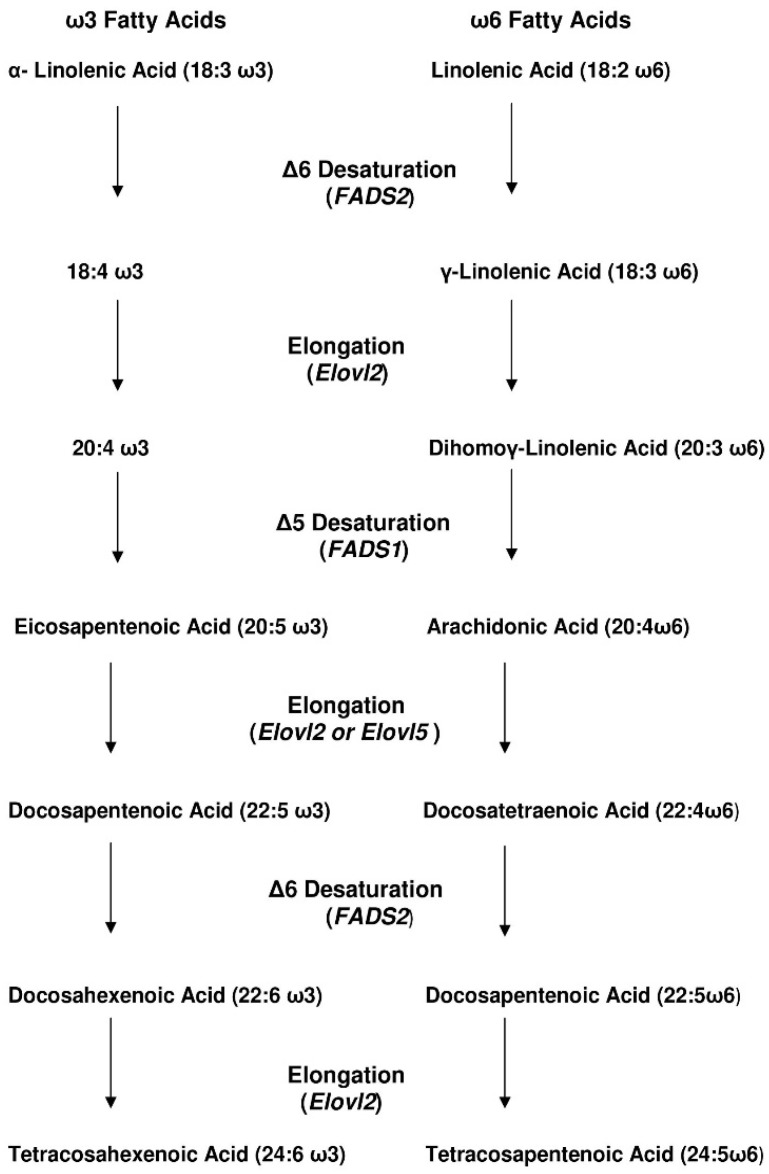
Linoleic (C18:2*n*-6) and α-Linolenic (C18:3*n*-3) acid metabolism and elongation.

More than 80 years ago, Burr *et al.* [1] described the essential fatty acids (EFA) linoleic acid (ω6 LA) and alpha linolenic acid (ω3 ALA) in animals. Their research contributed to the knowledge and concept that normal growth, development and health in mammals depends on EFA nutritional supply. Both EFA share enzymes like the Δ6 desaturase and elongases, and, thus, compete as substrates in this metabolic pathway (Figure 1). The genetic variants of desaturases determine the nutritional requirements for the supply of fatty acids and consequently, health and/or disease, as previously reviewed [2,3].

During the course of mammalian development, fatty acids (FA) are transferred to the fetus through the placenta [4] and their composition depends, to a great extent, on the maternal diet [5]. Maternal nutrition during pregnancy-lactation can induce significant changes in body composition, physiology and metabolism in offspring. Thus, the Fetal Origins Hypothesis was inspired by evidence showing that adult cardiovascular disease begins through developmental activation of a set of genes and metabolic pathways in the offspring in response to *in utero* under- or over-nutrition [6]. FAs play a primary role in growth and development and it is now accepted that imbalances in their intake during pregnancy and lactation may result in permanent changes that affect appetite control, neuroendocrine function and energy metabolism in the fetus; thus, influencing the metabolic programming [7]. However, the roles of EFAs and the mechanisms by which they impact the long-term health of the offspring remains to be determined [8]. The importance of the FA composition in the diet and the significant differences between ALA and its metabolic products DHA and EPA has been spotted both in adults [9] and in maternal diets during pregnancy [10]. Some recent reviews even reinforce the importance of maternal dietary FA quality for the health outcomes in offspring [7,8,11,12].

Beyond the existing differences between human and animals, the basic tissue, physiological and morphological placental developments are conserved between species. Therefore, animal models can be considered suitable when researching the impact of nutritional FAs on development and their long-term influence on the offspring’s susceptibility to metabolic diseases, including obesity, insulin resistance (IR), and cardiovascular risk [13]. Importantly, when looking for a comprehensive understanding of the role of nutritional FAs on development for the prevention of adult disease, a nutrigenetics approach is recommended [14,15].

## 2. Dietary ω3 Fatty Acids and Health

DHA and EPA: As defined by Burr [1], ALA is the only ω3 essential FA for mammals, while DHA and EPA are its downstream metabolic products. The benefits of consumption or supplementation of ω3 polyunsaturated fatty acids (PUFAs) by adults on the prevention and treatment of obesity, metabolic syndrome (MS), and cardiovascular disease (CVD) have been reported [16,17]. Most of the reviewed studies were randomized-controlled intervention trials suggesting that supplementation with ω3 PUFA might improve some obesity-associated metabolic syndrome features such as insulin resistance, hypertension and dyslipidemia by decreasing plasma triglycerides [18]. Similarly, they also confer cardio protection by lowering blood pressure and through their benefits on vascular and anti-inflammatory properties [19]. The efficacy of ω3 PUFA on reducing myocardial infarction, arrhythmia, cardiac and sudden death, or stroke is, however, controversial [20]. Although it is now widely accepted that DHA and EPA have beneficial effects on CVD, it is not yet clear if these benefits are directly or exclusively related to DHA and EPA [21,22]. Other benefits of DHA and EPA, such as reversion of neuropathies, have also been described [23,24] for both plant and marine ω-3 FAs.

ALA: The relationship between ALA and chronic disease is unclear. As supported by human studies [22,23,24], high intake of ALA is protective against fatal ischemic heart disease. In later years, the use of ALA-rich oils deserved attention in the search for nutritional ways of preventing or ameliorating cardiovascular disease and metabolic syndrome. Rodriguez-Leyva *et al.* reported that epidemiological randomized studies using flaxseed oil as a preventive intervention in a healthy population or in subjects identified as "at risk" for CVD are missing [25]. Baxheinrich *et al.* reported the beneficial effects of ALA rich rapeseed oil on body weight, systemic inflammation and endothelial function in patients with MS traits [26,27]. These past works showed that providing ALA significantly contributed to reductions in systolic blood pressure, total cholesterol, LDL-cholesterol and insulin levels after six months. Moreover, ALA was shown to significantly decrease body fat mass, as well as improve both vascular function and inflammation. 

The effects of ALA, as well as those of EPA and DHA, on the metabolic syndrome have been further reviewed by Poudyal *et al.* [28]. They addressed ALA, DHA and EPA as individual entities, and provided evidence of potentially independent effects for each of the ω3 FAs on cardiovascular health. These same authors also reported that the three ω3 FA could each reduce inflammation in cardiac fibrosis and hepatic steatosis in a high-fat diet induced model of metabolic syndrome in rats. Those effects were associated to a complete suppression of Stearoyl CoA desaturase (SCD1) function. In those studies, ALA induced comparatively different FA redistribution of retroperitoneal fat, skeletal muscle and liver. Furthermore, it was suggested that the accumulation of the ω3 FA in adipose tissue, as well as in skeletal muscle, may account for their crucial role in the reduction of abdominal fat, inflammation, dyslipidemia and IR [28]. Conversely, recommendations for ALA intake in pregnant women for the prevention of metabolic diseases in offspring are limited [29,30,31,32,33].

## 3. How Efficient Is ALA Conversion to EPA and DHA in Humans?

Dietary ALA is metabolically converted into acetate or CO_2_ through β-oxidation, or desaturated and elongated into EPA, ω3 DPA and DHA [34]. ALA conversion to longer products in tracer studies has been observed in nearly all humans studied from birth through late middle-aged men and women [34,35]. 

It is clear that the metabolism of ω3 FA depends on other nutrients, in particular ω6 FAs, due to their competition for the same enzymes and transport systems [2]. They also compete for incorporation into more complex lipids that comprise mammalian tissues, where high levels of ω6 PUFA replace and reduce ω3 PUFA levels. Early studies of rat liver microsomes showed that the Δ-6 desaturase activity measured *in vitro* was subject to competitive inhibition by other substrates. In particular, desaturation of ALA to ω3 eicosatrienoic acid was shown to be inhibited by LA and, conversely, LA conversion to ω6 gamma linolenic acid was inhibited by ALA [36,37]. 

When studying the impact of ALA on the improvement of the metabolic syndrome, the following question arises: is the conversion of ALA into EPA and DHA responsible for the observed effects? This question was addressed by Truong *et al.* [32] in relation to genetic variants in the Δ6 desaturase gene (*Fads2*). Adipose tissue was used as a biomarker of ALA intake in adult humans, and the authors showed that high concentrations of ALA in adipose tissue were associated with lower prevalence ratios of the metabolic syndrome compared to low ALA. Although an interaction between ALA and *Fads2* genotype (T-del) was borderline significant, it nevertheless suggested that genetic variation may play an important role along with diet in the development of metabolic syndrome, at least in the studied population. A variety of models have confirmed that ALA accumulates significantly and is converted into longer ω3 FA in humans [34]. According to the ISSFAL official statement #5, studies in healthy adults showed that supplementing ALA to Western diets containing LA raises DHA and EPA levels in blood and breast milk [38]. In addition, these and other studies have provided evidence indicating that FA accumulation is tissue-dependent [38] suggesting that metabolism may be based upon a tissue-selective need for longer ω3 FA, such as DHA.

As claimed by Anderson *et al.* [39], clarification of ALA’s involvement in health and disease is essential. Indeed, it is insufficient to assume that ALA exerts its beneficial effects through conversion to EPA and DHA. More thorough research is required to identify the differential effects of ALA on metabolic disease like IR and CVD and to differentiate the possible heterogeneous effects of ALA *versus* DHA and EPA. The use of developed animal models such as the Δ6 knockout mouse [40] that inhibits the conversion of ALA into EPA, and EPA into DHA, could be highly useful in this regard. No such work has been performed to date.

## 4. Adults

It has been observed that humans of all ages, from preterm and even fetuses to adults, convert ALA to DHA. However, the efficiency of conversion seems to decrease as infants mature [35]. ALA is partitioned to β-oxidation as energy source, for metabolic conversion to longer PUFA and for incorporation into tissues [39,41,42]. Importantly, studies were also reported in which a significant increase in plasma DHA levels was achieved by altering the oils in the diet and changing both ALA and LA content [43].

There seems to be an agreement that the partitioning of ALA towards β-oxidation in humans is lower in women than in men [44,45], an effect attributed to estrogen [34], which may explain the higher conversion of ALA to longer-chain PUFA in women [32,41]. The explanation for the preferred use of ALA for β-oxidation seems to be the greater affinity of carnitine palmitoyl transferase-1, the rate limiting enzyme in mitochondrial β-oxidation, for this EFA compared to other PUFA [41]. Most studies examining ALA have been performed in young men. The few studies focused specifically on women of reproductive age showed that conversion of ALA to EPA and DHA is 2.5× greater in women compared to age-matched men [43,44,45]. This is ascribed not only to the differential partitioning towards β-oxidation, but also to an up-regulation of the translocation of very long metabolic products towards the peroxisome in women [34]. It has been suggested that ALA conversion increases in pregnancy, which is supported by data in pregnant rats [44,45,46].

Clearly, gender differences exist and are of importance when recommending ω3 FA to men or women and, in this case of the latter, whether they are pregnant or not [41]. Work performed by Childs and others [46,47] have provided further support for this concept. The examination of whether there are sex differences in the long-chain ω3 FA response to increased ALA intake in humans showed that women have a higher increase than men in the EPA content of plasma phospholipids after six months. The gender differences in ALA use, metabolism and destination, warrant further investigation.

## 5. Fatty Acids Quality and Early Life

Pregnancy is supposed to be a period of high requirement for DHA in humans due to the fetus’ need for rapid growth and neural development [34]. FA levels in the embryo and newborn babies are directly associated with maternal FA levels and composition; therefore, any variation in the maternal intake of FAs susceptible to genetic variability is pivotal for fetal growth, development and health [47]. There is some evidence suggesting that the influence of genetic variation in *FADS* genes on both circulating and tissue FA profiles, which can influence disease risk, may have a trans-generational effect [48,49]. Moreover, it has been shown that breastfeeding also exposes babies to the maternal genetic Fads variations through their effects on milk quality and quantity of fatty acids that, in turn, affect the baby health and intelligence quotient [50]. Consequently, these studies show that the influence of *FADS2* polymorphisms in the mother is of uttermost importance for the array of FAs transferred from mother to child during uterine development and breastfeeding. This knowledge reinforces the importance of a nutritional adjustment during the critical perinatal period.

DHA and EPA: The effects of different qualities of dietary FAs during pregnancy and/or lactation on fetal development and offspring metabolism have been recently reviewed [7]. Maternal consumption of diets rich in ω3 PUFA in particular showed benefits for the development of offspring and it was even suggested that they exert epigenetic regulation for the prevention of obesity, insulin resistance and cardiovascular diseases [51,52]. The consumption of DHA and EPA during crucial periods of fetal development has beneficial physiologic and metabolic effects on the health of offspring by protecting them from the onset of metabolic diseases [7,11]. Nevertheless, results in the field are controversial, and the independent effects of ALA have not been studied enough.

ALA: During pregnancy there is a reduction of DHA in maternal serum while, at the same time, an increased requirement of DHA and EPA for fetal brain development [38,49]. The solution to compensate for this supposed imbalance can be to either provide ALA, the precursor for DHA and EPA, or these end products directly. Not enough scientific information exists comparing for the benefits of either supplementation. From the clinical aspect, criticism is raised regarding the claim of support for fetal cognitive health and brain function improvement associated with DHA and EPA supplementation [53]. 

Larque *et al.* [12] reported a somewhat positive relationship between maternal or cord serum DHA percentages and cognitive skills in young children. Unfortunately, valuable information was missing from this study: ALA is not mentioned amongst ω3 FAs and the blood FA composition of mothers and/or children was not described.

During the literature search described here, only one study was found indicating a negative effect of maternal over-consumption of ω3 FAs on life span and auditory brainstem response in older adult offspring. As in several papers, the term ω3 FA was broadly used, without specifying the dietary FA composition and without mentioning the specific effects of ALA [54].

## 6. Perinatal Manipulation of ALA

In relation to perinatal metabolism, significant FA desaturase activities have been detected in fetuses and preterm infants, indicating the existence of the molecular pathways at early stages of life [55,56].

Published research regarding the positive effects of DHA and EPA has been reviewed showing that they are essential for proper fetal development [57]. Supplementation of ω3 PUFA during pregnancy has been linked to decreased incidence of allergies [58]. An interesting study using flaxseed oil in mice questions whether ALA provision during gestation and lactation could induce epigenetic changes in maternal and offspring livers [58]. In this study, the authors described an interaction between ALA dietary content and the FA metabolism, through down-regulation of the expression of enzymes involved in the elongation (*Elovl2*) and desaturation (*Fads1*) of FAs in maternal livers. A positive correlation between *Fads2* promoter methylation in maternal livers and offspring livers with changes in the expression of DNA methyltransferases at day 19 of pregnancy was described. Even though the work was inconclusive, the authors suggested that the maternal availability of ALA during gestation - lactation could differentially alter the metabolism of ω3 and ω6 FAs, as well as the epigenetic status of *Fads2* in maternal and offspring’ livers. 

Research focusing on ALA content in the maternal diet and the long-term side effects on metabolic syndrome in the offspring is scarce. Our laboratory has begun focusing on those effects and showed that maternal dietary enrichment of ALA, compared to SFA, led to lower body weight gain, liver fat accumulation, and homeostasis model assessment (HOMA) index, as well as reduced SCD1 in the adult offspring exposed to a high fat diet [59]. Those results suggested that the relative increase in dietary ALA during pregnancy and lactation may have the potential to prevent obesity and insulin resistance in the offspring. Furthermore, Shomonov-Wagner *et al.* [60] compared the effects of dietary enrichment in ω3 ALA, DHA or EPA, compared to saturated fatty acids (SFA). That work showed that SFA, independent of total fat amount or calories, induced liver lipid accumulation and IR in offspring at weaning, while ALA was the most efficient ω3 FA to prevent the induced metabolic alterations. That work proposed that not only ALA and SFA have divergent effects on IR and liver lipid levels, but also that each of ALA, DHA and EPA behaved differently. Furthermore, ALA preventive effects were, apparently, unrelated to its conversion into DHA and EPA. Consequently, we conclude that ω3 ALA, DHA and EPA should be studied and referred to individually and not as a group. 

Recently published results seem to indicate that ALA, as opposed to SFA, up-regulates the expression of genes involved in lipid oxidation and in the circadian rhythm [61].

## 7. Regulatory Mechanisms

The ω3 FA family seems to have distinct abilities to modulate metabolic functions and gene expression [62]. Deckelman *et al.* [63] mentioned that the longer chain length and higher number of double bonds give these FAs unique properties and suggested that they relate to modulation of enzymes associated with signaling pathways/incorporation of EPA and DHA into membrane phospholipids and direct effects on gene expression, amongst others. However, ALA has not been studied enough, and the molecular interactions between ALA and genes, are only now beginning to be described [61].

Maternal dietary enrichment with ALA and the long terms effects on offspring susceptibility to obesity and metabolic syndrome are an important and urgent subject waiting to be investigated. Also important to consider are FA-gene interactions. Based on the literature found, we may assume that ALA has beneficial effects during gestation, presumably on the prevention of obesity-associated disease in offspring [61]. Ahmed [64] described a proteomics approach to examine the regulatory roles of ω3 FAs. By using mice fed high or low ω3 FA containing diets, some affected proteins were identified related to lipid, glucose and protein metabolism. Unfortunately their work excluded ALA, but their information hints at the interaction between FAs and protein targets for the regulation of metabolic pathways. These results, together with preliminary evolving information, warrant more studies that would benefit from testing ALA, DHA and EPA separately.

## 8. Epigenetics

Environmental factors such as diet during fetal development can induce long-term modifications in the genes of the fetus. Human epidemiological data and controlled animal studies corroborate the impact of diet in the perinatal period and its lasting effect on gene expression and metabolism [65]. The question, “how do FAs influence the establishment of an epigenotype” [66], is intriguing and stimulating. It is proposed that the peroxisome proliferator-activated receptor alpha (PPARa), an abundant nuclear receptor transcription factor, may be a candidate to be regulated by maternal dietary FAs in embryonic life. Wang *et al.* [67] described the way maternal and, possibly, paternal imbalanced over- and under-nutrition may induce epigenetic modifications. Thus, DNA methylation, histone modification and miRNAs, may regulate genome activity and gene expression leading to proteins that affect fetal programming and organ physiology with lifelong consequences, sex-dependent in some cases. The developmental adaptations that permanently change structure, physiology and metabolism in offspring would thereby predispose for metabolic and cardiovascular risk later in adult life. Vickers’s [68] analyzed not only the *in utero,* but also the trans-generational, possibilities for epigenetic modifications and its consequences. Niculescu *et al.* [58] showed epigenetic modifications induced specifically by ALA in an *in vitro* study. ALA was shown to alter the distribution of cells by influencing cell cycle phases, apoptosis and gene and protein expression of DNA methyl transferases 1 and 3. However, no modifications in global DNA methylation were found.

Burdge *et al.* [69] reviewed the interaction between FAs and the epigenome to conclude that it is not known, at present, how FAs modify the epigenome. Some of the existing limitations include the lack of definition of the experimental diets and the susceptibility of histone deacetylases to inhibition by short chain FAs present in most experimental diets. Nevertheless, the mechanisms underpinning transmission of developmental programming urgently require further research.

## 9. Final Thoughts and Recommendations

The importance of ω3 FAs for the development and long-term health of offspring is widely recognized. The importance of ALA, specifically, is only now beginning to be recognized; however, more thorough research is necessary in order to better understand its independent role in developmental programming.

Based on ongoing research [28,61] we propose here the concept that ALA may have a role in the programming of health. Specifically, it may have intrinsic regulatory properties on gene expression during fetal development that extend beyond its simple metabolic conversion to DHA and EPA.

Although the human conversion of ALA to DHA and EPA is gender-related and relatively low (up to 4%) [41], a higher consumption of ALA related to LA may increase it. Besides, individual DHA and EPA are not easily available and expensive. Moreover, DHA- and EPA-rich fish oil has some health disadvantages [22] due to contaminating factors such as heavy metals, teratogens, and others. 

Due to the widespread use of the general term “ω3 FA”, that is sometimes misleading, we propose that scientific publications apply a more precise nomenclature to identify the specific FAs tested (*i.e.*, ALA, DHA, and EPA).

In order to enlarge the understanding of ALA’s role in fetal development and programming, we recommend to analyze the effects of a maternal diet enriched in each of the individual ω3 FAs, in simple nutritional animal models that examine the tissue distribution, as well as gene expression and metabolic outcomes, in offspring.

## Abbreviations

ALA	ω3 alpha linolenic acid
FA	fatty acid
CVD	cardiovascular risk disease
DHA	docosahexenoic acid
DPA	docosapentenoic acid
EFA	essential fatty acid
EPA	eicosapentenoic acid
*Fads2*	gene of Δ6 desaturase enzyme
IR	insulin resistance
LA	linoleic acid
LDL	low density lipoproteins
Ppargc1a/*Ppargc1a*	peroxisome proliferative activated receptor gamma coactivator1a enzyle and gene, respectively
PUFA	polyunsaturated fatty acid
SCD1/*Scd1*	stearoyl-CoA desaturase 1 enzyme and gene, respectively
SFA	saturated fatty acid
